# Low-dose human menopausal gonadotrophin versus natural cycles in intrauterine insemination for subfertile couples with regular menstruation

**DOI:** 10.1186/s13048-020-00638-3

**Published:** 2020-04-04

**Authors:** Sichen Li, Yuxia He, Mingzhu Cao, Hanyan Liu, Jianqiao Liu

**Affiliations:** 1 Department of Reproductive Medicine, the Third Affiliated Hospital of Guangzhou Medical University, 63 Duobao Road, Guangzhou, Guangdong China; 2 Key Laboratory of Reproductive Medicine of Guangdong Province, Guangzhou, Guangdong China; 3 Key Laboratory for Major Obstetric Diseases of Guangdong Province, Guangzhou, Guangdong China; 4 Key Laboratory of Reproduction and Genetics of Guangdong Higher Education Institutes, Guangzhou, Guangdong China

**Keywords:** Intrauterine insemination, Ovarian stimulation, Clinical pregnancy rates, Live birthrates, Twin pregnancy

## Abstract

**Background:**

Women with irregular menstruation should be considered to benefit from the ovarian stimulation. However, most literature did not separate ovulatory disorders from normal menstrual cycles. Our purpose was to assess the superiority of ovarian mild stimulation compared with the natural cycle in IUI for subfertile couples when the women with regular menstruation.

**Methods:**

A retrospective study in a single medical center in which 2413 couples with 3573 IUI cycles were studied from 2013 to 2018. The results of IUI in natural cycles versus low-dose HMG induced cycles were analyzed.

**Results:**

For young women (age < 35 years) with normal menstrual cycle, HMG induced ovulation combined with IUI can improve clinical pregnancy outcome (13.55% in two follicular induced cycles vs. 7.23% in natural cycles, *p* < 0.01); even if only one follicle was induced, the clinical pregnancy rate was increased to 10.32% (*p* < 0.01). When two growth follicles were induced in HMG cycles, a remarkable improvement of the live birthrate (10.28% vs. 5.91% in natural cycles, *p* < 0.05) was noted. Simultaneously, twin pregnancy rates were increased to 20.69% (*p* < 0.01). Twin pregnancies showed significantly increased risk of both ectopic pregnancy and preterm birth (*p* = 0.00 for both). For advanced women (age ≥ 35 years) with regular menstrual cycle, ovulation induction didn’t improve clinical pregnancy and live birthrates, while age was the only relevant factor.

**Conclusions:**

Combining HMG induced ovulation and IUI can improve pregnancy outcome in young women with normal menstrual cycles. 1–2 follicles with diameter ≥ 14 mm served as the purpose of ovulation induction. Further, both twin and ectopic pregnancy rate in HMG cycles with two growth follicles were significantly higher than those in natural cycles were. Therefore, doctors must evaluate the risk before making choices and inform the patients to achieve the best results. For advanced women with normal menstrual cycles, natural IUI cycles were optional.

## Introduction

One in seven heterosexual couples suffers from infertility [1]. Intrauterine insemination (IUI) is considered as a routine therapy of subfertility in most clinical treatments. It was first described in 1962 [2]. The basic principle of IUI treatment is to improve the pregnancy rates by allowing most of healthy sperm to reach the fertilization site. IUI is simple and easy with fewer risks and manipulation. Looking into the direct health care costs in the same cohort of patients, IUI proved to be the most economical strategy for couples with unexplained or mild-male factor subfertility [3, 4].

IUI has a wide range of indications, containing all kinds of unexplained infertility and minor and mild endometriosis. Mostly, IUI or induced ovulation/IUI is helpful since most of the infertility causes may be unknown or untreatable [5]. Promoting IVF or ICSI with immediate effect of getting pregnancy cannot substitute the role of IUI for cases with unexplained and mild-male factor subfertility, especially considering the cost-effectiveness of IUI [6, 7]. IUI combined with ovarian stimulation are an alternative if IVF is not available immediately.

Ovarian stimulation can bring about modest results in IUI therapy. The drawbacks are low success rate and high risk of multiple births. The treatments of small follicles (12-14 mm) on trigger day have significant impact on multiple pregnancy rates in IUI cycles [8]. Our goal is to gain a satisfactory pregnancy rate and an acceptable multiple pregnancies outcome. The potential side effects of ovarian stimulation can be relieved by the low-dose rule. Low-dose gonadotropins are often used for ovarian stimulation. Gonadotrophins are glycoprotein hormones that regulate gonadal development and promote sex hormone production and secretion. Urine Human menopausal gonadotropin (HMG) is a cheap and effective drug commonly used in IUI therapy. An RCT showed the results of low-dose HMG induced IUI cycles were a higher clinical pregnancy rate (HMG 14.4% vs. CC 9.0%), higher live birthrate (HMG 13.8% vs. CC 8.7%) and lower number of preovulatory follicles (HMG 1.2 vs. CC 1.5, *P* < 0.001) [9]. Recombinant FSH (rFSH) can also be used for mild ovarian stimulation in women with unexplained infertility [10]. In the cases of similar clinical outcomes, IUI cycles induced by rFSH needed a shorter duration and a lower total gonadotropin dose [11, 12].

It is believed that one out of every three oocytes gained from ovulation induction is of high quality [13]. Therefore, to improve the pregnancy rate while reducing ovarian hyperstimulation and multiple pregnancies, the following three principles should be followed for ovulation induction in IUI. (1) Control the number of initiation follicle and growth follicle. (2) Control the ovulation number of dominant follicles. (3) Achieve ovulation of 1 or 2 dominant follicles each ovulation cycle. Multi-follicular growth is in connection with increased pregnancy rates in IUI combined with controlled ovarian hyperstimulation (COH). Multiple pregnancy rates were increased in three or four follicular cycles, but the overall pregnancy rate did not improve significantly. Therefore, IUI combined with COH was not recommendable for more than two follicles. One induced follicle may be the safest, and two follicles were acceptable by careful clinical consultation [14]. Further researches on IUI treatment with mild stimulation (1–2 follicles) are desirable.

Women with irregular menstruation are thought to benefit from the ovarian stimulation. However, most literature did not separate ovulatory disorders from normal menstrual cycles. Our purpose was to assess the superiority of ovarian mild stimulation compared with the natural cycle in IUI for subfertile couples when the women with regular menstruation. Most patients with regular menstruation cycle have normal ovulation. Whether ovulation induction of single follicular development is helpful or not in such patients compared with the natural cycle in IUI? There are fewer researches about pregnancy outcome when one or two follicles (≥14 mm) grow up respectively. Therefore, we conducted this retrospective study.

## Materials and methods

### Patients

We retrospectively analyzed the data of 2413 couples in our center from June 2013 to September 2018. There were 3573 IUI cycles in total. The patients were divided into two groups: the young women group (aged 2134 years old) and the advanced women group (aged 3546 years old). All the young and aged women were divided into three groups respectively: natural cycle, one growth follicle undergoing HMG induction, two growth follicles undergoing HMG induction. Inclusion criteria were: non-PCOS (polycystic ovary syndrome), with regular menstruation (21-35 days), 1–2 dominant follicles. Exclusion criteria were: patients with unruptured follicles 2 days after IUI, abnormal and anovulatory cycles, luteinized unruptured follicles (LUFs), PCOS, twice IUI in a single cycle, sexual dysfunction, artificial insemination by donor semen.

### Treatment

Ovarian stimulation, semen optimization and insemination were processed according to our protocols [15]. We use HMG to control ovarian stimulation. Transvaginal ultrasound checking was performed on cycle day 35 to exclude ovarian cysts larger than 30 mm. According to the women’s age, AMH, BMI and AFC, the patient was prescribed to start daily intramuscular injections of HMG (Livzon, Zhuhai, China). Injection continued until ovulation of at least one follicle≥17 mm in diameter. The doses of HMG ranged from 37.5 IU to 75 IU. The patients with more than two follicles larger than 14 mm in diameter were excluded.

The Trigger criteria: (1) The leading follicle was more than 17 mm in diameter. (2) The serum LH was elevated and the leading follicle was at least 14 mm in diameter. (3) Serum P concentrations≥1.5 pg/l and the leading follicle was at least 14 mm in diameter. If one of the three standards was met, we could trigger ovulation with hCG (5000–10,000 IU).

Insemination was performed 12-36 h after hCG injection. The semen was collected by masturbation after 2–7 days of sexual abstinence. Semen preparation was limited within 1H after ejaculation by density-gradient centrifugation. The semen volume used for insemination varied between 0.2 mL and 0.5 mL.

Rupture of dominant follicle after IUI was detected by transvaginal ultrasonography. We clarified the presence of free fluid in Douglas pouch and visible corpus luteum and/or disappearance of the follicle.,The patients whose dominant follicle had not ruptured 2 days after IUI were excluded. Cycles with at most two-follicular growth (14 mm) were used for data analysis to remove the high-order multiple implantations.

After insemination, micronized progesterone (200 mg vaginal capsule, twice a day) was used for luteal support. The serum β-HCG level was tested for pregnancy about 14 days later.

The main target was clinical pregnancy rates, the live birthrates, miscarriage rate, and multiple pregnancies rate were also considered. Clinical pregnancy referred to the presence of a yolk sac at 7 weeks of gestational by transvaginal ultrasound examination. Live birth referred to a surviving newborn. Miscarriage was defined as no vital or visible loss of pregnancy at transvaginal sonography. The clinical pregnancy rate was given each inseminated cycle but not each started cycle. Live birth and clinical pregnancy rate were calculated using each IUI cycle as the denominator.

### Statistical analysis

SPSS, version 25.0 software was used for statistical analysis. Continuous variables were tested for normal distribution by the One-SampleKolmogorov-Smirnov Test. For the variables showing skewed distribution, the Kruskal-Wallis H Test was used first to compare three groups of samples, then Mann-Whitney U Test was applied for two-sample comparisons. *P*-value < 0.017 was considered statistically significant. When the variables had a normal distribution, the t-test was used to compare two samples. Categorical variables were expressed as frequency (percentage) and compared using chi-square test. Multivariate logistic regression analysis was used to assess the relation between ovarian induction and pregnancy results. A *p*-value< 0.05 was considered to have a remarkable difference (t-test, Kruskal-Wallis H Test and chi-square test). A confidence interval (CI) of 95% was applied where suitable.

## Results

Two thousand four hundred thirteen couples and 3573 treatment cycles were contained in this study. There were 1716 (48.03%) natural cycles, 1569 HMG induced cycles with one growth follicle (43.91%) and 288 HMG induced cycles with two growth follicles (8.06%). Patient demographics are presented in Table 1. There were no statistically significant differences in the maternal age, antral follicle count (AFC) and AMH respectively between natural and ovulation induction cycles (*P* > 0.017). The difference between HMG cycle and the natural cycle was significant when maternal body mass index (BMI) was≤18.4 (*P* < 0.05).

**Table 1:** Baseline characteristics of the sample population stratified by ovulation induction method

Characteristic	Natural cycle (*n* = 1716)	one growth follicle in HMG cycle (***n*** = 1569)	two growth follicles in HMG cycle (*n* = 288)	one growth follicle vs. natural ***P*** value	two growth follicles vs. natural *P* value	Differences between three groups *P* value
**Maternal age (year)**	32.00	31.00	31.00	0.033^*^	0.106^*^	0.057
≤34(%)	1286(74.94)	1201(76.55)	214(74.31)	0.29	0.826	
≥35(%)	430(25.058)	368(23.45)	74(25.69)			
**Maternal Body Mass Index (kg/m2)**	20.50	21.00	20.20	0.00^*^	0.059^*^	0.00
≤18.4(%)	282(16.44)	213(13.58)	56(19.51)	0.022	0.345	
18.5–24.9(%)	1301(75.86)	1182(75.38)	213(74.21)			0.505
≥25(%)	132(7.70)	173(11.03)	18(6.27)			0.468
**AFC**	15	15	16	0.133^*^	0.229^*^	0.133
**AMH**	3.59	3.92	3.22	0.289^*^	0.592^*^	0.505
**endometial thickness (mm) on the day of hCG administration**	8.78 ± 1.76	9.18 ± 1.90	9.51 ± 1.83	0.000	0.000	
**Estrogen on the day of hCG administration**	1090.32 ± 409.628	1430.56 ± 623.69	2404.88 ± 1023.90	0.000	0.000	
**mean dose of HMG (IU)**		553.11 ± 428.64	637.06 ± 455.92			
**Duration of infertility (year)**	3.21 ± 1.89	3.11 ± 1.73	3.69 ± 2.26	0.639	0.185	
**Infertility**						
primary infertility (%)	1053(61.36)	976(62.21)	182(63.19)	0.640	0.600	
secondary infertility (%)	663(38.64)	593(37.79)	106(36.81)			
**Infertility diagnosis**				0.890	0.075	
Unexplained (%)	737(42.95)	665(42.38)	115(39.93)			
Cervical infertility (%)	3(0.17)	3(0.19)	3(1.04)			
Semen factor (%)	889(51.80)	812(51.75)	155(53.82)			
Endometriosis (%)	87(5.07)	89(5.67)	15(5.208)			

Continuous variables with normal distribution were expressed as mean ± SD, whereas continuous variables with a skewed distribution were reported as the median that include age, BMI, AMH and AFC. The variables for the three groups were compared by Kruskal-Wallis H Test. * represents the *p* value of the comparison between each of the two medians of the three groups using the Mann-Whitney U Test. *P* < 0.017 was considered statistically significant. Categorical data were expressed as frequency (percentage) and processed using Chi-square test. *P* < 0.5 was considered statistically significant when using T/Chi-square/ Kruskal-Wallis H test.

The pregnancy outcomes of IUI are showed in Table 2. Higher clinical pregnancy rates were showed in the HMG induced cycles of both one (9.75%, *P* < 0.05) and two (11.46%, *P* < 0.05) follicles compared with the natural cycles (127/1716, 7.4%). The live birthrate was increased in HMG induced cycles, but there was no difference or improvement compared with the natural cycles in either single or two follicular developments. Simultaneously, HMG induction brought about the risk of twin pregnancy. When two growth follicles were induced, the twin pregnancy rates were up to 18.18% (0% in natural cycles, *p* < 0.01). The ratio of miscarriage was increased from 12.90% in natural cycles to 23.13% in one growth follicle group (*P* < 0.05). In two-growth follicles group, the risk of ectopic pregnancy was raised (12.12% vs. 2.36% in natural cycles, *P* < 0.05).

**Table 2:** Pregnancy outcomes stratified by ovulation induction method

Characteristic	Natural cycle (*n* = 1716)	one growth follicle in HMG cycle (*n* = 1569)	two growth follicles in HMG cycle (*n* = 288)	one growth follicle vs. natural *P* value	two growth follicles vs. natural *P* value
**Clinical pregnancy**	127(7.40%)	153(9.75%)	33(11.46%)	0.016	0.020
**Live birth**	105(6.12%)	112(7.14%)	24(8.33%)	0.240	0.158
**Intrauterine Pregnancy**	124	147	31		
**Miscarriage**	16(12.90%)	34 (23.13%)	7(22.58%)	0.033	0.181
**Ectopic pregnancy**	3(2.36%)	6(3.92%)	4(12.12%)	0.466	0.028
**Twin pregnancy**	0(0)	1(0.65%)	6(18.18%)	1.0	0

For women younger than 35 years old, HMG induced cycles may cause an apparently higher clinical pregnancy rate than the natural cycles (*P* < 0.01). Clinical pregnancy was observed in 124 of 1201 induced cycles with one growth follicle (10.32%) and 93 of 1286 natural cycles (7.23%) at an odds ratio (OR) of 1.48 (95% CI, 1.11–1.96). In two growth follicles group, the clinical pregnancy rate reached 13.55% and significantly different from that in natural cycles (OR = 2.01, 95% CI = 1.29–3.14). The ratio of live birth (*p* < 0.05) was improved to 10.28% corresponding to an odds ratio of 1.82 (95% CI, 1.11–3.00) compared with the natural cycle. The twin pregnancy rate was increased from 0% in natural cycles to 20.69% (*p* < 0.01) (Table 3).

**Table 3:** Pregnancy outcomes stratified by ovulation induction method for maternal aged< 35 years old

Characteristic	Natural cycle (*n* = 1286)	one growth follicle in HMG cycle (*n* = 1201)	two growth follicles in HMG cycle (*n* = 214)	one growth follicle vs. natural *P* value OR (95%CI)	two growth follicles vs. natural *P* value OR (95%CI)
**Clinical pregnancy**	93(7.23%)	124(10.32%)	29(13.55%)	0.007 1.48 (1.11–1.96)	0.002 2.01 (1.29–3.14)
**Live birth**	76(5.91%)	94(7.83%)	22(10.28%)	0.06 1.35 (0.99–1.85)	0.018 1.82 (1.11–3.00)
**Intrauterine Pregnancy**	90	118	28		
**Miscarriage**	12(13.33%)	23 (19.49%)	6(21.43%)	0.033 (1.060–3.891)	0.181 (0.730–5.310)
**Ectopic pregnancy**	3(3.23%)	6(4.84%)	3(10.34%)	0.558 (0.371–6.266)	0.142 (0.659–18.182)
**Twin pregnancy**	0	1(0.81%)	6(20.69%)	1.0	0

The data of multivariate binary regression analysis for the woman under the age of 35 were showed in Table 4. Compared with the natural cycle, there was a positive correlation in HMG-induced cycle about the clinical pregnancy results, regardless of one (*p* < 0.05) or two follicles (*p* < 0.01). The negative correlation factors of clinical pregnancy included: BMI low body weight, maternal age and duration of infertility (*p* < 0.05, respectively). The live birthrate of HMG induced cycle with one follicle was similar to that of the natural cycle. HMG induced cycle with two follicles had a positive correlation with live birthrate at an OR of 1.76 (95% CI, 1.06–2.93; *p* < 0.05). Long duration of infertility (*p* < 0.01) and low BMI (*p* < 0.05) also affected live birth. The live birthrate of women under 35 years old was not significantly correlated with age.

**Table 4:** Multivariate logistic regression (Maternal age < 35 years old)

Characteristic	Clinical pregnancy		Live birth	
	OR (95%CI)	P	OR (95%CI)	P
**Maternal age (year)**	0.95 (0.90–0.99)	0.025	0.95 (0.90–1.00)	0.060
**Maternal Body Mass Index (kg/m2)**				
18.5–24.9	reference			
≤ 18.4	0.61 (0.40–0.91)	0.017	0.55 (0.34–0.88)	0.012
≥ 25	1.28 (0.80–2.03)	0.301	1.02 (0.58–1.78)	0.947
**Duration of infertility**	0.92 (0.857–0.996)	0.040	0.88 (0.80–0.97)	0.007
**Ovarian stimulation**				
natural cycle	Reference			
one growth follicle in HMG cycle	1.41 (1.06–1.87)	0.018	1.29 (0.94–1.77)	0.113
two growth follicles in HMG cycle	1.95 (1.24–3.06)	0.004	1.76 (1.06–2.93)	0.030

For women≥35 years old, miscarriage rate was increased apparently when one follicle was induced with HMG (*p* < 0.05). The other parameters showed no obvious difference compared with the natural cycle group (Table 5).

**Table 5:** Pregnancy outcomes stratified by ovulation induction method for women≥35 years old

Characteristic	Natural cycle (*n* = 430)	One growth follicle in HMG cycle (*n* = 368)	Two growth follicles in HMG cycle (*n* = 74)	One growth follicle vs. natural *P* value OR (95% CI)	Two growth follicles vs. natural *P* value OR (95% CI)
**Clinical pregnancy**	34 (7.91%)	29 (7.88%)	4 (5.41%)	0.988 0.996 (0.595–1.670)	0.454 0.665 (0.229–1.933)
**Live birth**	29 (6.74%)	18 (4.89%)	2 (2.70%)	0.270 0.711 (0.388–1.302)	0.197 0.384 (0.090–1.645)
**Intrauterine Pregnancy**	50	16	66		
**Miscarriage**	4 (11.76%)	11 (37.93%)	1 (33.33%)	0.020 4.58 (1.27–16.57)	0.322 3.750 (0.27–51.37)
**Ectopic pregnancy**	0 (0)	0 (0)	1 (25%)		
**Twin pregnancy**	0	0	0		

Multivariate dual regression analysis for advanced women aged≥35 years (Table 6) showed the live birthrate was only negatively correlated with age (*p* < 0.05). After controlling for age and the other factors, there were no statistically significant differences in live birthrate and clinical pregnancy rate between induced cycles and natural cycles.

**Table 6:** Multivariate logistic regression (maternal age ≥ 35 years old)

Characteristic	Clinical pregnancy		Live birth	
	OR(95%CI)	P	OR(95%CI)	P
**Maternal age (year)**	0.92 (0.82–1.03)	0.160	0.83 (0.71–0.97)	0.016
**Maternal Body Mass Index (kg/m2)**				
18.5–24.9	reference			
≤ 18.4	1.78 (0.85–3.71)	0.126	0.94 (0.35–2.51)	0.909
≥ 25	1.59 (0.82–3.02)	0.177	1.39 (0.65–2.99)	0.397
**Duration of infertility**	0.97 (0.87–1.06)	0.474	1.01 (0.91–1.11)	0.889
**Ovarian stimulation**				
natural cycle	reference			
one growth follicle in HMG cycle	0.99 (0.59–1.67)	0.968	0.67 (0.37–1.24)	0.206
two growth follicles in HMG cycle	0.70 (0.24–2.04)	0.515	0.39 (0.09–1.66)	0.201

## Discussion

In this study, we compared the utility of mild ovarian stimulation with natural cycle in IUI for couples with normal regular menstruation cycle and an intermediate prognosis. We reported the 5-year IUI practice (3573 cycles) and the doctor’s selective preference in the Third Affiliated Hospital of Guangzhou Medical University. We aimed to provide a safer and more efficacious reference scheme for doctors.

Ovulation induction was used to obtain mature follicles so that patients had a normal chance of conceiving. If the patients with unexplained infertility could ovulate normally, the number of dominant follicles can be increased appropriately after ovulation induction. It can shorten the time of pregnancy test and increase the probability of pregnancy. Compared with natural cycle, IUI combined with ovulation induction can significantly improve the clinical pregnancy rate and the live birthrate of women aged< 35 years with unexplained infertility [16, 17]. The meta-analysis of the Cochrane database drew a similar conclusion that the clinical pregnancy rate in ovulation induced IUI was about 2 times than in the natural/IUI cycle [18]. Conversely, an RCT discovered that ovulation induced IUI had no benefit for couples with unexplained subfertility and a moderate prognosis [19]. Our results indicated that the clinical pregnancy rate was increased remarkably by HMG induced ovulation (*p* < 0.05). Another research concluded that the clinical pregnancy rate of single follicular development group was about 6.45%, while the group with more than two dominant follicles was about 14.55% [20]. Tomlinson et al. found that the shift in the IUI cycle from a single follicle to double follicles may increase the pregnancy by 3.4 times [21]. Instead, there was no significant difference about pregnancy rates between single and multiple follicular growth cycle, no matter the growth follicle with an average diameter of at least 15 mm or 10 mm [19]. Zeng et al. concluded that the success rate of one dominant follicle induced and natural IUI cycle was similar [22]. In our study, clinical pregnancy rate rose in either one or two follicle induction cycles. Therefore, we only need one or two follicular growth to obtain a good pregnancy outcome of ovarian stimulation/IUI.

Although IUI is considered as the preferred therapy for subfertility at present, it is not an ideal substitute for IVF treatment. The higher multiple pregnancy rates contrast to the single-child pregnancy mainly in human fertility. It was demonstrated that the number of intermediate follicles (from 12 to≤15 mm) was an independent and significant risk factor for multiple pregnancies during r-FSH induced IUI cycle (*p* = 0.002 and *p* = 0.007) [23]. In our center, follicles≥14 mm on HCG day were selected as the growing follicles, and the multiple pregnancy rates were significantly reduced. No OHSS happened and the safety was good. Therefore, follicles≥14 mm on HCG day were selected as our standard for grouping in this study. In the ovarian stimulation cycle, IUI with high-dose gonadotropin rule (≥150 IU) was corresponding to twins and higher-order pregnancy rates of 28.6 and 8.2%, respectively. In our study, we used low-dose gonadotropin (the starting dose was 37.5–75 IU) for ovarian stimulation to reduce the potential adverse effect such as multiple pregnancies. The multiple births rate was comparable to the natural cycle in low-dose scheme. Our result suggested that one growth follicle in HMG cycle was recommended. The twin pregnancy rate was increased from zero in the natural cycle group to 0.65%, but the difference was not significant (*p* > 0.05). The twin pregnancy ratio was 18.18% in two growth follicles group and differed remarkably with the natural cycle group (*p* < 0.01). Besides, ectopic pregnancy rate was raised in the two growth follicle cycles (12.12% vs. 2.36% in natural cycles, *p* < 0.05). The follow-up results showed that twin pregnancy were more likely to have ectopic pregnancies than singleton pregnancy (*P* < 0.01, Table S1). The risk of multiple births in IUI with 2–3 follicles must be far less than that of 2–3 embryos transfer during IVF cycles. There is no comparative study to clarify the relative risk of matching follicles and embryos. For women aged < 35, although the twin pregnancy rate was as high as 20.69% (*p* < 0.01), the clinical pregnancy rate (13.55% vs. 7.23%, *p* < 0.01) and live birthrate (10.28% vs. 5.91%, *p* < 0.05) in HMG induced cycles with two growth follicles was significantly higher than the natural cycle, respectively. Therefore, doctors must assess the risk in advance to make a choice in order to achieve the best outcome. Spontaneous abortion and cesarean section rates were not significantly different between twin and singleton pregnancies (*P* > 0.5, Table S2, S3). However, the rate of preterm births in twin pregnancies was significantly higher than that in single pregnancies (*P* < 0.01, Table S3). Further observation and research were needed as the number of twins was relatively small.

After age 35, fertility declines and conception time increases [24–27]. The number of senior women who want to have children is increasing after adopting two-child policy in China. IUI is one of the treatments for senior subfertile women. A small cohort study of 262 IUI cycles combined with gonadotropins induced COH found that advanced women had a poor prognosis after COH or IUI [28]. Our study proved that COH with gonadotropins could not improve the clinical pregnancy or live birthrates for the older women in either one or two induced follicles/IUI compared with natural/IUI. Age was the unique high-risk factor for the advanced women. Yin et al. also revealed that ovulation induced IUI treatment did not significantly promote the clinical pregnancy for women over 35 years old [29]. Induced ovulation of either one or two follicles can increase the women’s blood estrogen and endometrial thickness, thereby changing clinical pregnancy and live birthrate, with aging particularly after 35 years old. The risk factor of age highlights a more important role, even overlooks the advantages of the follicle number from ovarian stimulation. A randomized controlled study of advanced women with unexplained infertility compared the COH/IUI cycles with immediate IVF cycles. The results showed that in the immediate IVF group, the pregnancy rates were higher and the treatment cycles were fewer [30]. Therefore, for older women with normal menstrual cycles, natural/IUI cycle is optimal and the immediate IVF is considerable. Many studies had confirmed that more than 80% of pregnancies happened within the first 3 cycles [16, 31]. They believed that if there was no pregnancy after three IUI cycles, especially in advanced women, IVF might be considered. A randomized controlled study showed that the live birthrate of three IUI treatment was no less than one IVF treatment [32]. Therefore, our patients underwent IUI for no more than 3 cycles.

In summary, induced ovulation in IUI can improve results of subfertile couples with regular menstruation by carefully controlling the risks of multiple pregnancies and OHSS. There may be some bias in the statistical results due to its retrospective design. A prospective, randomized controlled study is needed for further evaluation. Meanwhile, the prognostic factors of IUI patients with non-PCOS infertility contain infertility cause and duration, age, lower-BMI, total active sperm and so on. In the face of complex conditions and individual differences, we need to conduct in-depth and targeted researches in the future.

## Conclusion

For young women (age < 35 years) with normal menstrual cycle, HMG induced IUI can improve clinical pregnancy outcome, even if only one growth follicle (follicle≥14 mm in diameter on HCG day at least). Ovulation of 2-growth follicles≥14 mm on HCG day induced by HMG can improve the live birthrate. Simultaneously the multiple birth rates are increased significantly. Twin pregnancies showed obviously increased risk of both ectopic pregnancy and preterm birth. For advanced women (age ≥ 35 years) with regular menstrual cycle, ovulation induction does not improve clinical pregnancy and live birthrate, while age is the unique reason affecting fertility. Therefore, HMG induced IUI in young women with normal menstrual cycle can improve pregnancy outcome and 1–2 follicles with diameter ≥ 14 mm serves as the purpose of ovulation induction. For advanced women with normal menstrual cycles, a combination of natural cycle with IUI is optional.

## Supplementary information

**Additional file 1: Table S1.** Clinical pregnancy (including intrauterine pregnancy and ectopic pregnancy) follow-up of twin and singleton pregnancy. **Table S2.** Intrauterine twin and singleton pregnancy (excluding ectopic pregnancy). **Table S3.**Follow-up of live birth in twin and singleton pregnancy.

## Data Availability

All data generated or analyzed during this study are included in this published article.

## Abbreviations

HMG: Human menopausal gonadotrophin
IUI: Intrauterine insemination
IVF: In vitro fertilization
OHSS: Ovarian hyperstimulation syndrome
RCT: Randomized controlled trial
COH: Controlled ovarian hyperstimulation
PCOS: Polycystic ovary syndrome
AFC: Antral follicle count
BMI: Body mass index
