# Supplementary material for: Low-dose human menopausal gonadotrophin versus natural cycles in intrauterine insemination for subfertile couples with regular menstruation
Source: J Ovarian Res. 2020 Apr 4;13:36. doi: 10.1186/s13048-020-00638-3 (PMC7129328; doi:10.1186/s13048-020-00638-3)
Supplement: Supplementary file 1 — Additional file 1: Table S1. Clinical pregnancy (including intrauterine pregnancy and ectopic pregnancy) follow-up of twin and singleton pregnancy. Table S2. Intrauterine twin and singleton pregnancy (excluding ectopic pregnancy). Table S3.Follow-up of live birth in twin and singleton pregnancy. [file 13048_2020_638_MOESM1_ESM.docx]

Table S1.Clinical pregnancy (including intrauterine pregnancy and ectopic pregnancy) follow-up of twin and singleton pregnancy

|  | Singletons(306) | Twins(7) | P value |
| --- | --- | --- | --- |
| **combined intrauterine and ectopic pregnancy** | 0 (0/306) | 14.29% (1/7) | 0.00 |
| **ectopic pregnancy** | 3.59% (11/306) | 0 (0/7) |  |
| **intrauterine pregnancy** | 96.41% (295/306) | 85.71% (6/7) |  |

Table S2.Intrauterine twin and singleton pregnancy (excluding ectopic pregnancy)

|  | Singletons (295) | Twins (7) | P value (Fisher's Exact Test) |
| --- | --- | --- | --- |
| **Spontaneous abortion** | 18.98% (56/295) | 14.29% (1/7) | 1.0 |
| **Live birth** | 81.02% (239/295) | 85.71% (6/7) |  |

Table S3.Follow-up of live birth in twin and singleton pregnancy

|  | Singletons (239) | Twins(6) | P value(Fisher's Exact Test) |
| --- | --- | --- | --- |
| **Delivery gestational age**  [Premature delivery](http://dict.youdao.com/w/premature%20delivery/#keyfrom=E2Ctranslation) (%) | 7.11% (17/239) | 83.33% (5/6) | 0.000 |
| [Full-term birth](http://dict.youdao.com/w/full-term%20birth/#keyfrom=E2Ctranslation) (%) | 92.89% (222/239) | 16.67% (1/6) |  |
| **Delivery way**  [Eutocia](http://dict.youdao.com/w/eutocia/#keyfrom=E2Ctranslation) (%) | 59.15% (139/ 239) | 50% (3/6) | 0.692 |
| [Cesarean delivery](http://dict.youdao.com/w/cesarean%20delivery/#keyfrom=E2Ctranslation) (%) | 40.85% (96/239) | 50% (3/6) |  |

Note: Preterm delivery was defined as delivery before 37 weeks gestation.
